# Increased prevalence of femoroacetabular impingement on the elderly with fractures of the proximal femur

**DOI:** 10.1051/sicotj/2021033

**Published:** 2021-05-20

**Authors:** Thiago Sampaio Busato, Marcelo Baggio, Marcelo Gavazzoni Morozowski, Gladyston Roberto Matioski Filho, Lucas Dias Godoi, Juan Rodolfo Vilela Capriotti

**Affiliations:** 1 Director of the Adult Hip Surgery Fellowship, CRIAr – Centro de Reconstrução e Instituto de Pesquisa Articular Curitiba 80540-220 PR Brazil; 2 Fellow of the Adult Hip Surgery Fellowship, CRIAr – Centro de Reconstrução e Instituto de Pesquisa Articular Curitiba 80540-220 PR Brazil; 3 Staff of the Adult Hip Surgery Group, CRIAr – Centro de Reconstrução e Instituto de Pesquisa Articular Curitiba 80540-220 PR Brazil; 4 Chair of CRIAr Group Curitiba 80540-220 PR Brazil

**Keywords:** Femoroacetabular impingement, Femoral neck fracture, Intertrochanteric fracture

## Abstract

*Objectives*: Femoroacetabular impingement (FAI) has been recently related to several pathologies, besides chondral injury and hip arthritis. We aim to investigate the prevalence of FAI morphology in an elderly cohort hospitalized due to a proximal femur fracture and compare these findings to a control group. We hypothesize that limited medial rotation due to FAI’s morphology could increase stresses to the proximal femur, acting as a facilitating mechanism for fractures in this region. Therefore, a higher prevalence of FAI morphology would be present in the study group. *Methods*: A retrospective cross-sectional study was performed based on the analysis of radiographic images in AP and lateral views of the fractured hip. Firstly, we have set to measure FAI prevalence in an elderly cohort victimized by fractures of the proximal by measures of the alpha, Tönnis, and lateral center edge angles of a hundred consecutive patients hospitalized for proximal femur fractures. Secondly, we have analyzed the possible relationship between the FAI subtypes and the type of fracture. Finally, we have compared this sample’s data with that of a similar control cohort not affected by fracture. *Results*: The cohort in this study displayed a higher prevalence of pathological changes in the Tönnis, center-edge, and alpha angles with odds ratios of 3.41, 2.56, and 4.80, respectively (with statistical significance). There was also a significant relationship between cam-type FAI and intertrochanteric fractures, corroborating our initial hypotheses. *Conclusions*: This study demonstrated that a cohort of older patients affected by fractures of the proximal femur had an increased prevalence of radiographic signs of femoroacetabular impingement. Furthermore, this is the first study demonstrating a statistically significant relationship of cam-type FAI with intertrochanteric fractures, suggesting a possible cause and effect relationship.

## Introduction

The femoroacetabular impingement syndrome (FAIS) is caused by the symptomatic conflict between the femur and the acetabulum [1, 2]. Proximal femur fractures are a public health issue in the older population and are a well-known risk factor for morbidity and mortality. While the association between female gender and low bone density are well described as risk factors for a proximal femur fracture, some recent studies highlighted the possible association of mechanical elements [3–5]. Faulknek et al. demonstrated that the hip axis length was higher in patients with proximal femoral fractures [6]. Additionally, Tokyay et al. [7] pointed out that acetabular morphology could predict the types of proximal femoral fractures among elderly patients. However, in the elderly population, there is an increased prevalence of some radiological signs of femoroacetabular impingement (FAI), such as coxa profunda (which by itself should not be considered as a pathological finding) [8–13]. FAI also has been linked to a variety of other injuries, such as stress fracture [14], avascular necrosis [15, 16], nonunion of femoral neck fractures [17], posterior traumatic dislocation [18], and pubalgia [19, 20].

**Figure 1:**
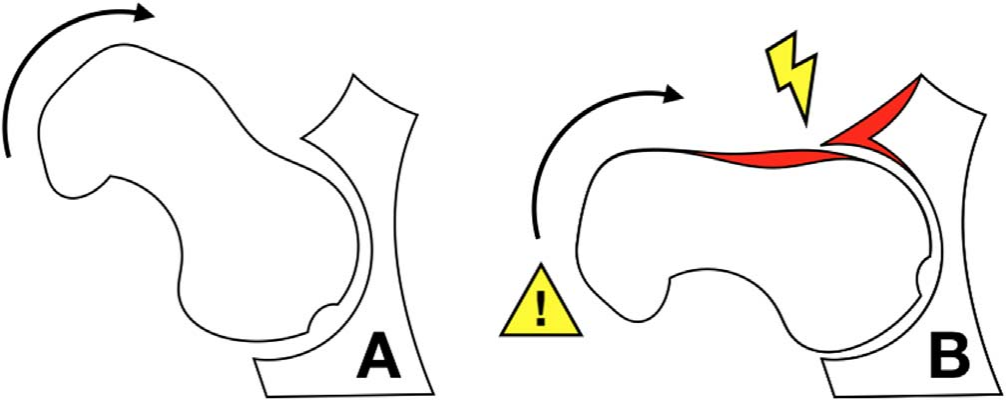
Illustration of the proposed fracture facilitating mechanism. In A, a hip with normal medial rotation may absorb the torsional force caused by indirect trauma. In B, a hip with limited internal rotation due to FAI morphology (red zones) would concentrate higher forces at the intertrochanteric area (exclamation sign).

Considering that the mechanism of torsional trauma has been implicated in the genesis of extra-capsular fractures of the hip [21] and that patients with FAI generally present a decrease in hip internal rotation [22, 23], it is fair to propose that the reduction in the range of motion, associated with a traumatic torsional force, could cause a sudden increase of stresses in the proximal femur, leading to a higher risk of fracture, especially on elderly patients with FAI morphology. The proposed mechanism is illustrated in Figure 1.

This study’s main objective was to identify the prevalence of radiographic signs of FAI in an elderly cohort affected by a fracture of the proximal femur. As secondary objectives, we have studied the relationship between the subtype of FAI and the type of fracture. Finally, we compared the findings to another previously studied control cohort to examine if there was a significant prevalence in these radiological signs.

## Material and methods

A retrospective cross-sectional study has been carried out by analyzing preoperative radiographic images from a hospital database in southern Brazil. The following inclusion criteria were used: acute fracture of the femoral neck or the trochanteric region, age over 60 years, and availability of the pelvis radiographic study in anteroposterior views and a lateral view of the affected hip. This study included patients selected consecutively to a pre-established number of a hundred hips between September 2014 and June 2019.

**Figure 2:**
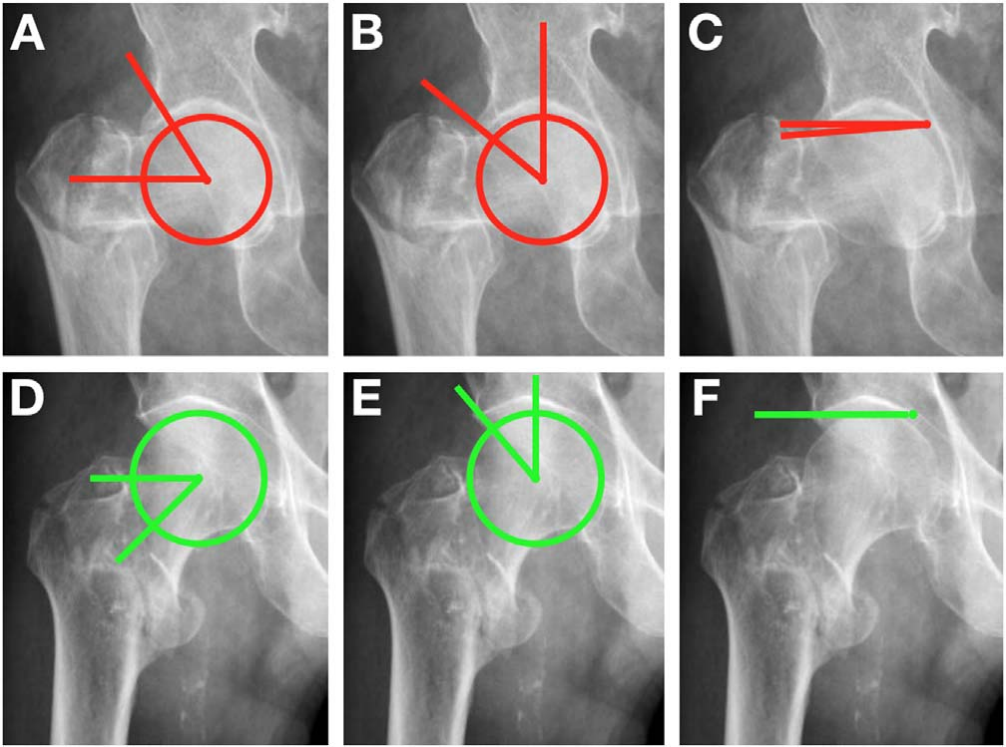
Two cases of the experimental group are illustrated. Top: AP view of a pathological FAI hip. A – alpha angle, B – lateral center edge angle, C – Tönnis angle. Bottom: AP view of a non-pathological FAI hip. D – alpha angle, E – lateral center edge angle, F – Tönnis angle.

The images have been retrieved for analysis and numbered in a database from 1 to 100. Next, we separated them into two groups: femoral neck fractures and intertrochanteric fractures. The following angles were measured on the affected side by a board-certified orthopedic surgeon. We adopted the following reference values for the definition of cam deformity: alpha angle > 55° [13] on AP or lateral view. The pincer deformity was defined as a lateral center edge (CE) angle > 40° or a Tönnis angle < 0° [24–26]. Pathological and normal angular values are exemplified in Figure 2. All the information from this group of individuals was collected from the hospital’s electronic medical record. We analyzed sex, age, fracture type, affected side, and evaluation of the measured angles. This cohort was then compared to a previous anthropometric tomographic sample, extracted from a similar population from a previous (in press) research carried out by the same institution.

The institution’s research ethics committee authorized the study under the number 3.723.119 registered at “Plataforma Brasil.”

The collected data were entered into spreadsheets (Excel) and then analyzed using SPSS (Statistical Package for the Social Sciences) 15.0 software. The data were studied using descriptive statistics. The qualitative variables were expressed through absolute numbers and frequencies, and the quantitative variables through standard deviation and frequency tables (simple and contingency). We verified the assumption of data normality through the Shapiro-Wilk test. To evaluate the relationship between two categorical variables, the Chi-square test was applied, and to compare two independent groups, the Mann Whitney test was used. The level of significance established was *p* ≤ 0.05.

One hundred hips were studied. Of the total sample, 63% were female, and 37% male. Patients had an average age of 75.72 (SD = 9.74) years, ranging from 60 to 98 years of age. The right hip was the most affected on the fracture group, covering 53% of the analysis. Of the total fractures, 77% had intertrochanteric involvement. The types of fractures did not differ in relation to the patient’s gender (*p* = 0.80) or age (*p* = 0.67). Mean age was similar in both groups, with a mean of 77.74 (SD = 10.06) years in the group with femoral fracture and 72.67 (SD = 8.42) years in the control group.

Sixty-six hips represented the control group, and the mean age of this sample was 72.67. Although there was a difference concerning the average age compared to the study group (77.74), this difference was irrelevant for this study.

## Results

### FAI prevalence

In this cohort, 49% of the patients with fractures of the proximal femur showed a pathological value of the alpha angle. Pathological measurements of the Tönnis angle were seen in 35%, and the center-edge angle was abnormal in 47% of these individuals.

The prevalence of pathological findings in the control group regarding alpha, Tönnis, and CE angle were respectively 16.67%, 13.64%, and 25.76%.

### Type of fracture and FAI

Of the 49 patients that presented pathological values of alpha angle on the experimental group, an intertrochanteric fracture was seen on 54.55% and a femoral neck fracture in 30.43%. Abnormal alpha angle showed a statistically significant relationship with intertrochanteric fractures (*p* = 0.042). There was no significant association with the type of fracture regarding abnormal values of center-edge angle (*p* = 0.39) or Tönnis angle (*p* = 0.31).

### Comparison between groups

On the fracture group, the mean alpha angle was pathological and substantially higher (57.40°) with *p* = 0.000006. In this group, the mean Tönnis angle (0.5°) was significantly lower (more aberrant) with *p* = 0.0006; and the mean center edge angle (41.38°) was also considerably pathologic with *p* = 0.000002. These results are summarized in Table 1.

**Table 1:** Mean values of alpha, Tönnis and center edge angle in both groups, minimum and maximum values, SD: standard deviation, and *P*-value (significant < 0.05).

	Experimental	Control	*P*-value
Alpha	57.40° (38 – 94, SD = 11.90)	48.73° (30 – 71, SD = 7.67)	0.000006
Tönnis	0.5° (−15 – 25, SD = 8.06)	3.05° (−12 – 50, SD = 7.54)	0.0006
Center edge	41.38° (25 – 62, SD = 8.08)	36.77° (30 – 60, 5.52)	0.000002

Regarding the alpha angle, the experimental group (49% abnormal) also displayed significant variation compared to the control group (16.67% abnormal), presenting an OR of 4.80 (*p* = 0.000001) when we compared the groups. A pathological Tönnis angle was also significantly higher (35% abnormal) than the observed in the control group (13.64% abnormal), with an Odds-ratio (OR) of 3.41 (*p* = 0.0041). Finally, the center edge was significantly more deviant than the average in the control group (47% versus 25.76%), with an OR of 2.56 (*p* = 0.0096).

## Discussion

Abnormal mechanical forces arisen from pathological proximal femur morphology have been recently implied as possible causative factors of fractures in this anatomic region [5, 14, 17, 18, 21]. Although the femoral neck’s direct impingement against the anterior acetabular rim mostly causes these forces, internal rotation’s inherent limitation also plays a role. Limitation of internal rotation is a common sign of FAI morphology related to abnormal stresses in the proximal femur [17, 18]. In their sample of patients who underwent arthroscopic repair of FAI, Polesello et al. presented a cohort with 100% of the cases displaying lower internal rotation degrees (IR) in the preoperative evaluation [22]. In another study, Hopkinson et al. suggest that the mechanism of extra-articular fractures of the proximal femur is related to torsional forces during a fall [21]. The present sample demonstrated that the increase in the alpha angle > 55° was statistically linked to trochanteric fractures. Thus, we can hypothesize that a torsional force, when transmitted to a hip with a limitation of internal rotation, could increase fracture risk. In the present study, we demonstrated a higher prevalence of radiographic signs of femoroacetabular impingement in elder patients victimized by proximal femur fractures and a positive relationship of CAM type FAI with intertrochanteric fractures.

The present study’s main limitation was the suboptimal radiographic technique. We did not strictly follow the criteria recommended for the FAI’s radiographic investigation in an elective setting because the radiographs were obtained in an emergency care scenario in patients with a painful fractured hip. For that reason, there may have been a loss in the diagnose of FAI in the studied series, but yet, ultimately favoring the null hypothesis. Besides that, the eventual presence of previous symptoms or other antecedents was also not explored due to the study’s retrospective nature. However, the radiograph technique may not have impaired the study results because, according to Tannast et al., the chosen measures would not change with different pelvic positions [26]. This study has considered the alpha angle above 55° as diagnostic of cam-type morphology and the center lateral edge angle > 40° or a negative Tönnis angle as representatives of pincer [10, 24, 25]. For this study’s purposes, the mixed type was studied into its separate components (cam or pincer type).

The threshold values for the measurements were based on previous studies. We considered the lateral center edge angle > 40° or a negative Tönnis angle as diagnostic of coxa profunda to decrease the over-diagnosing risk. The alpha angle threshold used was greater than 55°, which is the reference found in most studies that discussed this topic [3, 24, 25].

Beck et al. studied the relationship between FAI and femoral neck nonunion after osteosynthesis. They observed that patients with cam-type morphology were at higher risk of developing nonunion, depicting a possible connection between abnormal pathological forces affecting the fracture [17]. Yang et al. also associated the anterior impingement with fractures of the femoral neck in an analysis of 36 hips. They suggested that there could be a credible related mechanism for the fracture outcome, probably by the direct conflict of the femoral neck cortex against the anterior rim of the acetabulum [5].

The present research showed that patients with intertrochanteric fractures had an increased alpha angle with an average of 58.55° (*p* = 0.042). The control group yielded only 16.67% of pathological values in the alpha angle analysis, against 49% in the fracture group. In the control group, only nine hips (13.64%) displayed changes concerning the Tönnis angle (against 35% of pathological changes of the same measurement in the experimental group), with *p* = 0.0041. The statistics also confirmed that, concerning the center-edge angle, the experimental group had a prevalence of 47%, compared to 27.76% in the control group (*p* = 0.0096). Finally, the odds ratio for having an abnormal alpha, Tönnis, and center-edge angles value on the fractured hip were respectively 4.80, 3.41, and 2.56 (*p* ≤ 0.05).

Collectively, this data suggests that patients in the experimental group had a greater probability of generating the outcome (fracture) than the control group, which confirms the present study’s hypothesis. We believe that more research is still needed to elucidate better the data found in this study. However, it may indicate that having a previous FAI correction could eventually be revealed as a protective factor against proximal femoral fractures in the elderly.

## Conclusion

This study demonstrated that a cohort of older patients affected by fractures of the proximal femur showed an increased prevalence of radiographic signs of femoroacetabular impingement. Furthermore, this is the first study demonstrating a statistically significant relationship of cam-type FAI with intertrochanteric fractures, suggesting a possible cause and effect relationship.

## Conflict of interest

The authors declare that there are no conflicts of interest in this research.

## References

[1] Ganz R, Parvizi J, Beck M, Leunig M, Nötzli H, Siebenrock KA (2003) Femoroacetabular impingement: a cause for osteoarthritis of the hip. Clin Orthop Relat Res 417, 112–120. 10.1097/01.blo.0000096804.78689.c2 14646708

[2] Grantham WJ, Philippon MJ (2019) Etiology and pathomechanics of femoroacetabular impingement. Curr Rev Musculoskelet Med 12(3), 253–259. 10.1007/s12178-019-09559-1 PMC6684670 31278564

[3] Na Nakorn P, Tantigate D, Lertwanich P (2016) Prevalence of radiographic osteoarthritis and structural abnormalities of the hip in patients with contralateral hip fractures. J Med Assoc Thail 99(10), 1119–1125. 29952457

[4] Nardo L, Parimi N, Liu F, Lee S, Jungmann PM, Nevitt MC (2015) Femoroacetabular impingement: prevalent and often asymptomatic in older men: the osteoporotic fractures in men study. Clin Orthop Relat Res 473(8), 2578–2586. 25736918 10.1007/s11999-015-4222-0 PMC4488192

[5] Yang P, Fan H, Wang X, Xu S, Yang L, Chen G (2020) The association between anterior femoroacetabular impingement and femoral neck fractures: an observational study. Med (United States) 99(6), e19068. 10.1097/MD.0000000000019068 PMC7015654 32028429

[6] Faulknek KG, Mcclung M, Cummings SR (1994) Automated evaluation of hip axis length for predicting hip fracture. J Bone Miner Res. 9, 1065–1070. 7942153 10.1002/jbmr.5650090714

[7] Tokyay OA, Güven M, Encan ME, Okay E (2017) The influence of acetabular morphology on prediction of proximal femur fractures types in an elderly population. Hip Int 27(5), 489–493. 28574116 10.5301/hipint.5000476

[8] Mascarenhas V V, Rego P, Dantas P, Morais F, Mcwilliams J, Collado D (2016). European Journal of Radiology Imaging prevalence of femoroacetabular impingement in symptomatic patients, athletes, and asymptomatic individuals : A systematic review; 85:73–95. 10.1016/j.ejrad.2015.10.016 26724652

[9] Kuhns BD, Weber AE, Levy DM, Wuerz TH (2015) The natural history of femoroacetabular impingement. Front Surg 2, 1–7. 26636088 10.3389/fsurg.2015.00058 PMC4644807

[10] Nepple JJ, Lehmann CL, Ross JR, Schoenecker PL, Clohisy JC (2013) Coxa profunda is not a useful radiographic parameter for diagnosing pincer-type femoroacetabular impingement. J Bone Jt Surg 95(5), 417–423. 10.2106/JBJS.K.01664 23467864

[11] Clohisy JC, Baca G, Beaulé PE, Kim YJ, Larson CM, Millis MB (2013) Descriptive epidemiology of femoroacetabular impingement: a north american cohort of patients undergoing surgery. Am J Sports Med 41(6), 1348–1356. 23669751 10.1177/0363546513488861

[12] Diesel CV, Ribeiro TA, Coussirat C, Scheidt RB, Macedo CAS, Galia CR (2015) Coxa profunda in the diagnosis of pincertype femoroacetabular impingement and its prevalence in asymptomatic subjects. Bone Joint J 97-B(4), 478–483. 25820885 10.1302/0301-620X.97B4.34577

[13] Frank JM, Harris JD, Erickson BJ, Slikker W, Bush-Joseph CA, Salata MJ (2015) Prevalence of femoroacetabular impingement imaging findings in asymptomatic volunteers: a systematic review. Arthroscopy 31(6), 1199–1204. 25636988 10.1016/j.arthro.2014.11.042

[14] Goldin M, Anderson CN, Fredericson M, Safran MR, Stevens KJ (2015) Femoral neck stress fractures and imaging features of femoroacetabular impingement. PM R 7(6), 584–592. 25591871 10.1016/j.pmrj.2014.12.008

[15] Serong S, Haversath M, Jäger M, Landgraeber S (2019) Prevalence of cam deformity and its influence on therapy success in patients with osteonecrosis of the femoral head. J Tissue Eng Regen Med 13(4), 546–554. 30636359 10.1002/term.2794

[16] Fraitzl CR, Kappe T, Brugger A, Billich C, Reichel H (2013) Reduced head-neck offset in nontraumatic osteonecrosis of the femoral head. Arch Orthop Trauma Surg 133(8), 1055–1060. 23712709 10.1007/s00402-013-1771-0

[17] Beck M, Leunig M, Clarke E, Ganz R (2004) Femoroacetabular impingement as a factor in the development of nonunion of the femoral neck: A report of three cases. J Orthop Trauma 18(7), 425–430. 15289688 10.1097/00005131-200408000-00006

[18] Steppacher SD, Albers CE, Siebenrock KA, Tannast M, Ganz R (2013) Femoroacetabular impingement predisposes to traumatic posterior hip dislocation. Clin Orthop Relat Res 471(6), 1937–1943. 23423625 10.1007/s11999-013-2863-4 PMC3706669

[19] Strosberg DS, Ellis TJ, Renton DB (2016) The role of femoroacetabular impingement in core muscle injury/athletic pubalgia: diagnosis and management. Front Surg 3(February), 1–5. 26904546 10.3389/fsurg.2016.00006 PMC4751254

[20] Shetty VD, Shetty NS, Shetty AP (2015) Groin pain in athletes: a novel diagnostic approach. Sicot-J 1, 16. 27163072 10.1051/sicotj/2015017 PMC4849255

[21] Hopkinson-Woolley JA, Parker MJ (1998) Fractures of the hip: does the type of fall really affect the site of fracture? Injury 29(8), 585–587. 10209588 10.1016/s0020-1383(98)00133-8

[22] Polesello GC, Queiroz MC, Ono NK, Honda EK, Guimarães RP, Ricioli Junior W (2009) Tratamento artroscópico do impacto femoroacetabular. Rev Bras Ortop 44(3), 230–238. 27004177 10.1016/S2255-4971(15)30073-2 PMC4783678

[23] Said HG, Masoud MA, Morsi MMA-H, El-Assal MA (2019) Outcomes of hip arthroscopy for femoroacetabular impingement: the effect of morphological type and chondrolabral damage. Sicot-J 5, 16. 31115316 10.1051/sicotj/2019012 PMC6530372

[24] Mascarenhas VV, Ayeni OR, Egund N, Jurik AG, Caetano A, Castro M (2019) Imaging methodology for hip preservation: techniques, parameters, and thresholds. Semin Musculoskelet Radiol 23(3), 197–226. 31163499 10.1055/s-0039-1688714

[25] Barrientos C, Barahona M, Diaz J, Brañes J, Chaparro F, Hinzpeter J (2016) Is there a pathological alpha angle for hip impingement? A diagnostic test study. J Hip Preserv Surg 3(3), 223–228. 27583162 10.1093/jhps/hnw014 PMC5005062

[26] Tannast M, Fritsch S, Zheng G, Siebenrock KA, Steppacher SD (2015) Which radiographic hip parameters do not have to be corrected for pelvic rotation and tilt? Clin Orthop Relat Res 473(4), 1255–1266. 25231153 10.1007/s11999-014-3936-8 PMC4353539

